# The Metric System

**Published:** 1886-10

**Authors:** 


THE METRIC SYSTEM.—Prof. Oscar Oldberg, who has been noted as the most vigorous champion of the metric system of weights and measures, and to whose efforts the adoption of the system in the U. S. Pharmacopœia of 1880 was in a great part due, has recently published an article recanting what he has previously said in regard to this system and expatiating upon the superiority of the system now in vogue in America; the latter being quite as simple, universally understood, and much less liable to lead to mistakes in filling prescriptions. The latter point is the chief argument against the adoption of the metric system, the simple displacement of a dot being sufficient to cause a death when dangerous remedies are prescribed. The present system is one almost universally satisfactory, and Prof. Oldberg manifests his good judgment in abandoning his former position and coming forth as the exponent of the present system of prescription writing.—*Kansas City Medical Index.*

